# Strategies for molecular authentication of herbal products: from experimental design to data analysis

**DOI:** 10.1186/s13020-022-00590-y

**Published:** 2022-03-22

**Authors:** Hoi-Yan Wu, Pang-Chui Shaw

**Affiliations:** 1 Li Dak Sum Yip Yio Chin R & D Centre for Chinese Medicine, The Chinese University of Hong Kong, Shatin, Hong Kong, China; 2 School of Life Sciences, The Chinese University of Hong Kong, Shatin, Hong Kong, China; 3 State Key Laboratory of Research on Bioactivities and Clinical Applications of Medicinal Plants (The Chinese University of Hong Kong) and Institute of Chinese Medicine, The Chinese University of Hong Kong, Hong Kong, China

**Keywords:** Molecular authentication, Quality control, Herbal products, DNA metabarcoding, Genome skimming, Next-generation sequencing, Kraken, Genome2-ID

## Abstract

Molecular herbal authentication has gained worldwide popularity in the past decade. DNA-based methods, including DNA barcoding and species-specific amplification, have been adopted for herbal identification by various pharmacopoeias. Development of next-generating sequencing (NGS) drastically increased the throughput of sequencing process and has sped up sequence collection and assembly of organelle genomes, making more and more reference sequences/genomes available. NGS allows simultaneous sequencing of multiple reads, opening up the opportunity of identifying multiple species from one sample in one go. Two major experimental approaches have been applied in recent publications of identification of herbal products by NGS, the PCR-dependent DNA metabarcoding and PCR-free genome skimming/shotgun metagenomics. This review provides a brief introduction of the use of DNA metabarcoding and genome skimming/shotgun metagenomics in authentication of herbal products and discusses some important considerations in experimental design for botanical identification by NGS, with a specific focus on quality control, reference sequence database and different taxon assignment programs. The potential of quantification or abundance estimation by NGS is discussed and new scientific findings that could potentially interfere with accurate taxon assignment and/or quantification is presented.

## Introduction

DNA-based methods have already been adopted by various pharmacopoeias, including Chinese Pharmacopoeia [1–5], United States Pharmacopeia [6], British Pharmacopoeia [7, 8], Japanese Pharmacopoeia [9] and Hong Kong Chinese Materia Medica Standards [10], for herbal identification. The listed methods are all intended for identification of single-ingredient raw materials or “crude drugs” of natural products before manufacturing, but not intended for testing multi-ingredient samples. In reality, most traditional medicines involve the use of multiple herbs/ingredients in one treatment formula. There are a total of 96,592 formulae in the Dictionary of Traditional Chinese Medicine Formula, the largest and most comprehensive collection of Chinese medicine formula, and most of the listed formulae contain multiple ingredients. In Japan, there are 148 Kampo extract formulations approved and covered by national health insurance [11].

With the development of molecular techniques and next-generation sequencing (NGS), more and more studies on the molecular identification of multi-herb products have been published. The methods adopted by these studies can be generally classified into two approaches: (1) Sequencing-based identification and (2) Species-specific DNA marker detection. Species-specific DNA markers are developed based on single nucleotide polymorphisms or indels unique for the target species. The marker should also be conserved intra-specifically. Species-specific assays involving specific marker amplification(s) are usually highly specific, sensitive, and applicable to multi-ingredient matrices. They are robust, rapid with simple data analysis and low in running cost, as sequencing and subsequent sequence analysis are usually not needed. Moreover, real-time quantitative PCR (qPCR) and digital PCR (dPCR) are standard techniques in food testing industry. It would be straightforward to develop similar techniques for the identification of herbs. Recent researches demonstrated that quantification and semi-quantification of target species are possible by different molecular techniques, such as qPCR [12, 13], dPCR [14], vector control quantitative analysis [15] and double peak detection in nucleotide signature [16, 17], further expanding the scope of potential applications of this approach. However, species-specific assays cannot be used to identify unknown samples with no intended target species. They cannot detect allergens, pathogens, contaminants or adulterants that are unexpected and not included as one of the detection targets in the assay design. Sequencing-based identification, such as DNA barcoding, has the potential to obtain and detect the sequences of known and unknown, depending on the affinity of universal primers to template DNA [18] and the availability of the detected sequences in reference database for searching and comparison. However, conventional DNA barcoding relies on Sanger sequencing. If a PCR product contains multiple amplicons from more than one species, overlaying peaks would be obtained in the electropherogram, and the sequencing would be failed [18]. This issue can be partially solved by cloning the PCR products into a vector and sequence multiple clones individually, but the procedures are laborious and time-consuming. High throughput sequencing has drastically increased the sequencing efficiency and allows sequencing of millions of reads in a single run, presenting new opportunities for more in-depth analysis and simultaneous identification of multiple ingredients for quality control and pharmacovigilance. In this review, we are going to introduce the two major experimental approaches of using NGS for herbal identification, DNA metabarcoding and genome skimming/shotgun metagenomics, and discuss some important considerations in experimental design, reference database building, selection of bioinformatics analysis methods and the potential of quantification by NGS.

## General workflow of taxonomic identification by high-throughput sequencing

**Fig. 1:**
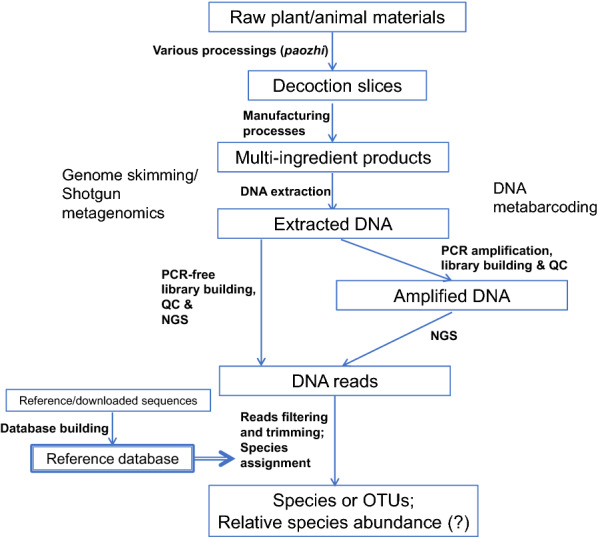
A flow diagram of species identification of multi-ingredient products by NGS

From raw plant/animal materials to multi-herb preparations/products, Chinese medicinal materials have undergone different processing procedures (*paozhi*) to become decoction slices, the processed herbal materials ready for making decoctions and products. Different decoction slices are then subject to further manufacturing processes to become multi-ingredient products. Figure 1 is a conceptual diagram showing species identification of multi-ingredient products by NGS. During various processing of raw herbs, DNA in the herb would have been fragmented and degraded. Filler (mostly plant-based) and excipients, such as rice, honey and ginger juice, would also be added, introducing additional sources of DNA. In NGS identification, good-quality DNA has to be extracted from the multi-herb products, in order to remove impurities or PCR inhibitors hampering subsequent library building or PCR amplification. Depending on quality and quantity of DNA obtained, as well as availability of bioinformatics pipeline and reference DNA database, the extracted DNA would be subject to different library preparation processes and experimental approaches, either DNA metabarcoding approach or genome skimming/shotgun metagenomics approach. In DNA metabarcoding approach, PCR would be carried out with universal primers to amplify barcode regions with good discriminatory power. PCR products should be purified, usually with Ampure XP beads (Beckman) [19], and quality-checked on Bioanalyzer (Agilent) or Tapestation (Agilent). Purified PCR products would then be subjected to library preparation, in which unique sample indexes and sequencing adaptors will be added to the PCR product. The kit and method used for library building depend on the sequencing platform to be used. After library construction, the library should be cleaned up and quality-controlled again. Quantification is also required for normalization of libraries belonging to different samples. Normalized libraries with different sample indexes can be pooled and sequenced together in the NGS platform. In genome skimming/shotgun metagenomics approach, total DNA from multi-herb products will be directly used to prepare the shotgun sequencing libraries after quantification. If more than 1 μg good-quality total DNA could be obtained, a PCR-free library building method, which involves DNA fragmentation (may not be necessary for herbal products with fragmented DNA), end repair, 3′ end adenylation and adaptor ligation, can be considered. Otherwise, an additional PCR step can be included using high-fidelity DNA polymerase. Similar to the metabarcoding approach, the library will then be cleaned up, checked for quantity and quality, normalized and pooled for high-throughput sequencing.

**Fig. 2:**
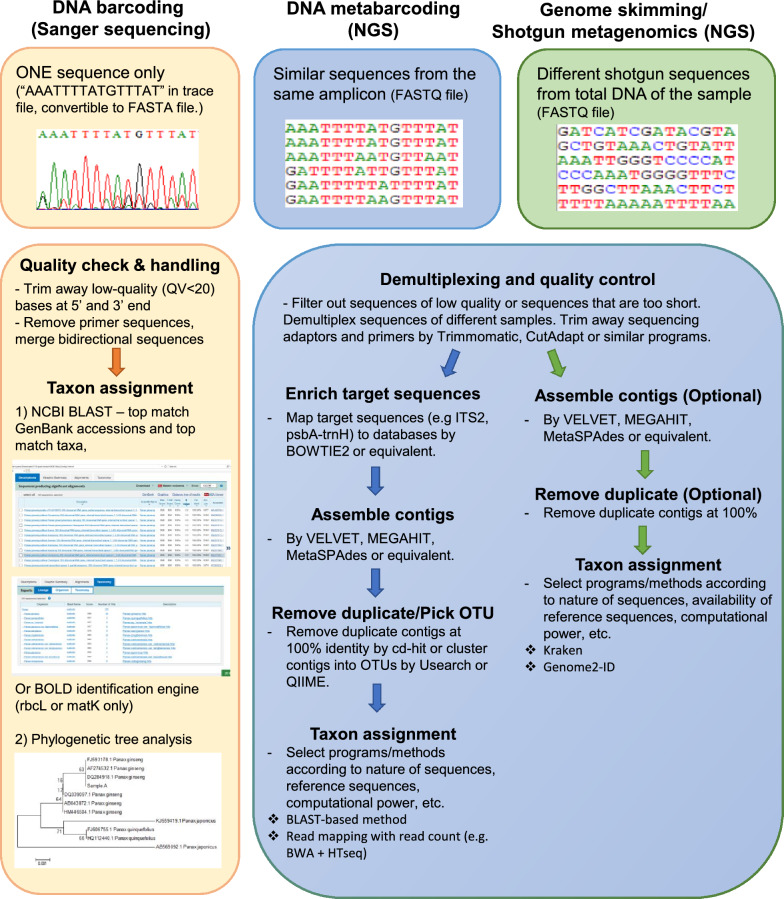
Sequences obtained and analysis workflow for DNA barcoding, DNA metabarcoding and genome skimming

After NGS, the sequencing reads will be subjected to bioinformatics analysis. Figure 2 shows the differences in sequencing results and subsequent analysis workflow between traditional DNA barcoding, DNA metabarcoding and genome skimming/shotgun metagenomics. For conventional DNA barcoding, only one sequence would be obtained from Sanger sequencing. After removing low-quality bases at 5′ and 3′ ends, BLAST search and phylogenetic analysis can be performed. For NGS-based approaches, raw reads would be pre-processed to trim away low-quality bases, sequencing adaptors and primers (if available), and then filtered to remove lengths that are too short or of low quality. For paired-end reads, it would be recommended to remove both ends if one end could not pass the filtration. Quality of pre-processed reads could be checked using software like FastQC [20]. Pre-processed reads could then be clustered into operational taxonomic unit (OTU) based on similarity at defined threshold (usually 99–100%), in order to reduce computation workload in taxon assignment analysis. Clusters containing very small number of reads (usually < 10) may be discarded to avoid false positive identification due to sequencing or PCR error. Representative consensus sequences from each cluster would then be subject to taxon assignment, usually by alignment-based identification like BLAST or k-mer-based methods like Kraken [21]. There are analysis platforms or packages, such as Galaxy [22] and QIMME 2 [23], that provide an end-to-end analysis pipeline with a wide range of tools/plugins to choose from. An overview on the workflow of DNA metabarcoding and subsequent bioinformatic analysis for herbal identification has been reviewed by Lo and Shaw [24].

## DNA metabarcoding

DNA metabarcoding is the combination of traditional DNA barcoding with high-throughput sequencing, allowing simultaneous sequencing of the same barcode amplicons from different species and the identification of multiple species within the same sample. It was first applied on the identification of Chinese Medicine products by Coghlan et al. [25], who identified 68 plant families from 13 multi-ingredient samples by amplification and high-throughput sequencing of the trnL c/h region. Since then, both second (mainly Illumina and Ion Torrent) and third (mainly PacBio and Nanopore) generation sequencing platforms have been used for authentication of various herbal medicines or herbal supplements or establishment of reference genome assembly of plant species [26–30]. This method is much more efficient than cloning amplicons for Sanger sequencing, and it is so sensitive that even DNA of filler can be sequenced and identified [27]. As PCR-based metabarcoding still relies on the amplification of barcode regions using universal primers, the success of identifying all ingredients within a sample is governed by the same factors that determine the discriminatory power of traditional DNA barcoding. (1) Heavy processing would degrade the DNA of the products, making amplification of common barcode regions with universal primers not possible; (2) Primer-template mismatch would lead to biased amplification or even lack of amplification for certain species, which could be a reason for non-detection of certain expected species [25, 31]; (3) Identification success still depends on the discriminatory power of the barcode selected. To overcome these limitations, metabarcoding of multiple mini-barcode loci on traditional medicines has been performed [32]. After comparing 12 different loci for 18 traditional medicines, Arulandhu et al. concluded that mini-barcode markers ITS2, mini-rbcL, trnL (P6 loop) and mini-16S were the most informative for identification of plants and animals in multi-ingredient traditional medicine. Yik et al. [33] combined adaptor ligation-mediated PCR with metabarcoding by ligating the total DNA extracted from processed herbal products to an adaptor, before carrying out PCR with one universal primer for psbA-trnH and one primer targeting the adaptor, such that short reads of psbA-trnH with variable lengths could be obtained during sequencing.

## Genome skimming/shotgun metagenomics

Genome skimming/shotgun metagenomics is the low-coverage shotgun sequencing of total DNA. Genome skimming of a single-ingredient plant material can provide sequences of high copy fraction of the genome, including nearly complete plastid genomes, nuclear ribosomal DNA, and partial kilobase-fragments of the mitochondrial genome [34]. When this approach is applied on herbal products, sequencing library is built without PCR amplification of barcode regions, circumventing the limitations of PCR in conventional DNA barcoding and DNA metabarcoding, such as limited number of barcode regions, DNA degradation during product manufacturing and PCR bias because of primer mismatch, etc. Table 1 summarizes the features and applicability of traditional DNA barcoding, DNA metabarcoding and genome skimming in herbal authentication. Ideally, it would be great to have a curated reference database built with genome skimming sequences of most plant species from expertly identified voucher specimens, similar to one suggested by Coissac et al. [35], to compare with the unknown sample. Building such database requires combined efforts of botanists, curators, molecular biologists and bioinformaticians, as well as a lot of resources. It is expected that discriminatory power of genome skimming method would be substantially higher than just focusing on traditional barcode loci, as the information content of whole-genome reference data set is much higher [36]. However, without PCR amplification, it would require a higher amount of good quality DNA extracted from the test samples for library building, which may not be easy to obtain from highly processed products. Currently, there are only a handful of publications reporting the application of shotgun metagenomics in the identification of herbal products. Handy et al. recently performed both DNA metabarcoding and genome skimming, complemented with HPLC–UV analysis, to evaluate 20 dietary supplements of *Echinacea* commercially available [21]. Using two different k-mer-based methods for taxon assignment, they reported that metabarcoding could only identify seven products to family level by Kraken2 while genome skimming could identify nine products up to species level and one product to genus level by Genome2-ID. In this work, rbcL and trnL P6 regions were selected for metabarcoding. Using more barcode regions might increase the rate of successful identification. Shotgun sequencing-based genome skimming collects sequences of total DNA within the sample. Depending on the abundance and coverage of the reference database used, more DNA regions could be exploited for taxon assignment.

**Table 1:** Features and applicability of different species identification approaches

	DNA barcoding	DNA metabarcoding	Genome skimming/shotgun metagenomics
Source of template	PCR product	PCR product	Total DNA
No. of sequences obtained	One	Thousands to millions	Thousands to millions
Read length	~ 1000 bp	Short (~ 100–300 bp) or long (> 10,000 bp), depending on sequencing platform	Short (~ 100–300 bp) or long (> 10,000 bp), depending on sequencing platform
Detection of multiple species	No	Yes	Yes
Affected by PCR bias	Yes	Yes	No
Potential for quantification	No	No (Read counting is possible but cannot truly reflect relative abundance)	Yes (Semi-quantification may be possible if all reads can be correctly assigned taxonomically)

Xin et al. sequenced the total DNA of Longdan Xiegan Wan, a ten-herb product with crushed powder of ten types of decoction slices moulded into pills, and filtered out the ITS2, psbA-trnH and matK sequences to BLAST in the DNA Barcoding System for Identifying Herbal Medicine [31]. They successfully identified all ten target species from the two reference mock products prepared in laboratory with authenticated decoction slices. Only six to seven target species and two common adulterant species were identified from three commercial product samples. However, these commercial products did meet the requirements of all test items documented in the Chinese Pharmacopoeia, revealing the limitations of current test items and requirements in ensuring the correct identity of all ingredients. Shi et al. sequenced the total DNA of three traditional Chinese medicinal products, Wuhu San [37], Qingguo Wan [38], and Fuke Desheng Wan [39], all of which were made of crushed powder of Chinese materia medica without further heating or chemical extraction. They enriched filtered reads belonging to target barcode regions, such as rbcL, matK and ITS2, with their python scripts [40] and assembled them into contigs. Duplicates of 100% identity were removed, and the contigs were clustered into OTUs for taxon assignment by BLAST search against several databases. This combination of shotgun sequencing and taxon identification using barcode sequences has been called shotgun metabarcoding [41]. By this means, Shi et al. identified not only all targeted species from the reference/mock product samples prepared in house, but also some known adulterants, weeds and fungi from commercial products. This method evades PCR bias in PCR-based metabarcoding and allows the use of abundant DNA barcode sequences as reference. This “targeted genome skimming” has shown the feasibility of identifying the biological ingredients in multi-ingredient herbal products by NGS without amplification. In our opinion, this approach has the potential of quantification, by establishing a correlation between the number of reads and biomass.

## Considerations in experimental design

### Nature of multi-herb samples/products

The degree of DNA degradation of the products depends on the manufacturing process. Treatments like heating, fuming or chemical extraction would lead to more serious DNA degradation. “Mild” treatments like powdering are less detrimental to the DNA and longer DNA fragments could be retained. For herbal powders, which are included in over 60% of the recorded traditional Chinese Patent Medicines in the Chinese Pharmacopoeia [31], PCR-based methods, such as metabarcoding and species-specific qPCR, are still applicable, as long as the length of the amplicons are not too long (less than 400–500 bp). Some decoction slices are already heavily processed. For example, red ginseng has been steamed and Rehmanniae Radix Praeparata has been stirred with yellow rice wine before steaming. PCR-based methods with long amplicons are not suitable for products containing heavily processed decoction slices. For heavily processed products, such as extracts or concentrated granules, metabarcoding with mini-barcodes or genome skimming could be considered.

### Setting up controls

Extraction blank control (EBC) should be set up during DNA extraction, working in parallel with other samples. For PCR-based method, absence of PCR products should be obtained from EBC. For genome skimming, ideally, the EBC should undergo library preparation and NGS together with other samples.

Reference mock herbal preparation should be prepared in duplicate or triplicate in the laboratory using decoction slices authenticated by experts/Chinese Medicine Pharmacists. Barcode sequences of each authenticated decoction slice should be obtained by PCR amplification and Sanger sequencing and included in the reference database for comparison during data analysis and taxon assignment. For metabarcoding, it is essential to carry out PCR for each decoction slice using the same primer sets for the amplification and library building. This is to ensure the amplifiability of each decoction slice using those primer sets.

Extraction positive control (EPC) should be a reference herbal material of a species not closely related to the species in the herbal product samples. It should have been shown to be amplifiable (for PCR-based metabarcoding) and identifiable using the analysis pipeline, with reliable reference barcode and organelle sequences in the reference database. It could be subjected to all experimental procedures from DNA extraction to NGS individually or be mixed with other authenticated decoction slices of the target species and made into one of the reference mock herbal preparations together. The EPC should be identified successfully during sequence analysis.

### Sequencing platform

Characteristics of various sequencing platforms have been well summarized by Lo et al. [24]. Generally speaking, Illumina sequencing platforms are lower in cost and have a lower error rate, but their maximum supported read lengths are short (up to 2 × 301 bp) [42]. PacBio Single-Molecule Real-Time (SMRT) sequencing and Nanopore sequencing allows real-time sequencing with much longer read lengths, up to 50 kbp for SMRT sequencing and up to 2.3 Mb for Nanopore sequencing [43]. Basecalling accuracy used to be a limitation of third generation sequencing platforms. However, it has been greatly improved in the past few years. The circular consensus sequencing (CCS) method developed by Pacific Biosciences allows generation of long reads (average length 13.5 kbp) with high accuracy (99.8%) [44]. A recent study compared the effects of sequence length on taxon classification accuracy using long (300–4000 bp) and short (100–300 bp) reads simulated based on known features of Illumina (short reads), Nanopore and PacBio (short and long reads) [45]. For short reads (100–300 bp) of plants and animals, Illumina reads had a higher recall (the ratio of correctly classified reads to all reads) than reads of Nanopore and PacBio. Increasing read length of Nanopore (2500–3000 bp) and PacBio (800–900 bp) could improve the recall and even surpass that of Illumina 300-bp reads in plants and animals. As herbal products usually contain short-length, degraded DNA, Illumina sequencing platforms with longer read length, 2 × 251 bp or 2 × 301 bp, would be suitable with generally higher sequencing throughput (more reads per run) than other platforms.

### Selection of barcode regions

Before deciding the target barcode regions to be amplified in metabarcoding, or to be mapped out and selected for analysis and comparison against a reference database in shotgun metagenomics, differentiation power of each barcode region for each listed species in the herbal product should be evaluated, preferably confirmed by phylogenetic tree analysis, using reference sequences of the target species and sequences of its closely related species. For metabarcoding, amplifiability of each listed species with the primers intended for library preparation should be tested on individual authenticated decoction slices.

### Reference sequence database

The importance of an accurate, reliable and suitable reference database cannot be overstated. The sequences that should be included in the reference databases depend on the target DNA regions to be involved in sequence analysis and taxon assignment. Ideally, all reference sequences in the database should be generated from voucher specimens authenticated by botanists/zoologists. The voucher specimens should be deposited in a herbarium/museum to establish sequence data traceability. This may not be feasible in reality, as the list of adulterants, substitutes or closely-related species of herbal medicines is by no means exhaustive. Currently, there are only a few curated sequence databases for taxonomic identification. The Barcode of Life Data System (BOLD) collects sequences from authenticated, well-recorded and vouchered samples [46]. The DNA Barcoding System for Identifying Herbal Medicine, also known as Traditional Chinese Medicine Database (TCMD) was built and curated by Institute of Chinese Materia Medica, China Academy of Chinese Medical Sciences [47]. It contains more than 78,000 barcode sequences from at least 23,000 medicinal species listed in the Chinese, European, Indian, Japanese, Korean and American Herbal Pharmacopoeias [48]. These databases only collect sequence data of a limited number of barcodes, and they may not be a suitable reference for identification of target species that cannot be differentiated form their closely related species using common DNA barcodes. They also have limited applicability for analysis of genome skimming data. More recently, Liao et al. has launched the Global Pharmacopoeia Genome Database [49], which is a mineable sequence database containing dozens of whole genome data sets, more than 23,000 complete plastid sequences (“superbarcodes”) and more than 200,000 DNA barcode sequences of traditional medicines from different international pharmacopoeias.

Most studies on the identification of herbal products involved custom databases with relevant sequences downloaded from GenBank, or direct BLAST search against nucleotide database of GenBank. Our recent study, however, revealed that the annotation of most barcode accessions of *Dendrobium* species are incomplete, and the taxonomic reliability of 7.14% evaluated barcode sequences were regarded as highly doubted [50]. There would be an intrinsic uncertainty based solely on GenBank nucleotide sequences without any further filtering or evaluation. This should be taken into account in subsequent taxon assignment analysis and validation. For instance, when only one or two sequences of an unexpected species in the reference database is matched by a small proportion of sample reads, the reliability of the reference sequence(s) of the unexpected species should be individually evaluated by BLAST or phylogenetic tree analysis. Another point to note is the completeness of the downloaded sequences after simple search with keywords. Searching for “psbA-trnH” or “trnH-psbA” sequences in GenBank nucleotide database would only output sequences of short fragments containing the psbA-trnH intergenic spacer. The psbA-trnH sequences in chloroplast complete genomes will not be included. It is because psbA-trnH sequence usually spans across the end and the beginning of a chloroplast genome. To obtain psbA-trnH sequences from chloroplast complete genomes to build a custom reference database of target regions, sequences after the trnH(GUG) gene and sequences before the psbA gene should be isolated and linked up. It may be preferable to include chloroplast complete genomes in the database.

### Sequence analysis and taxon/species assignment

**Fig. 3:**
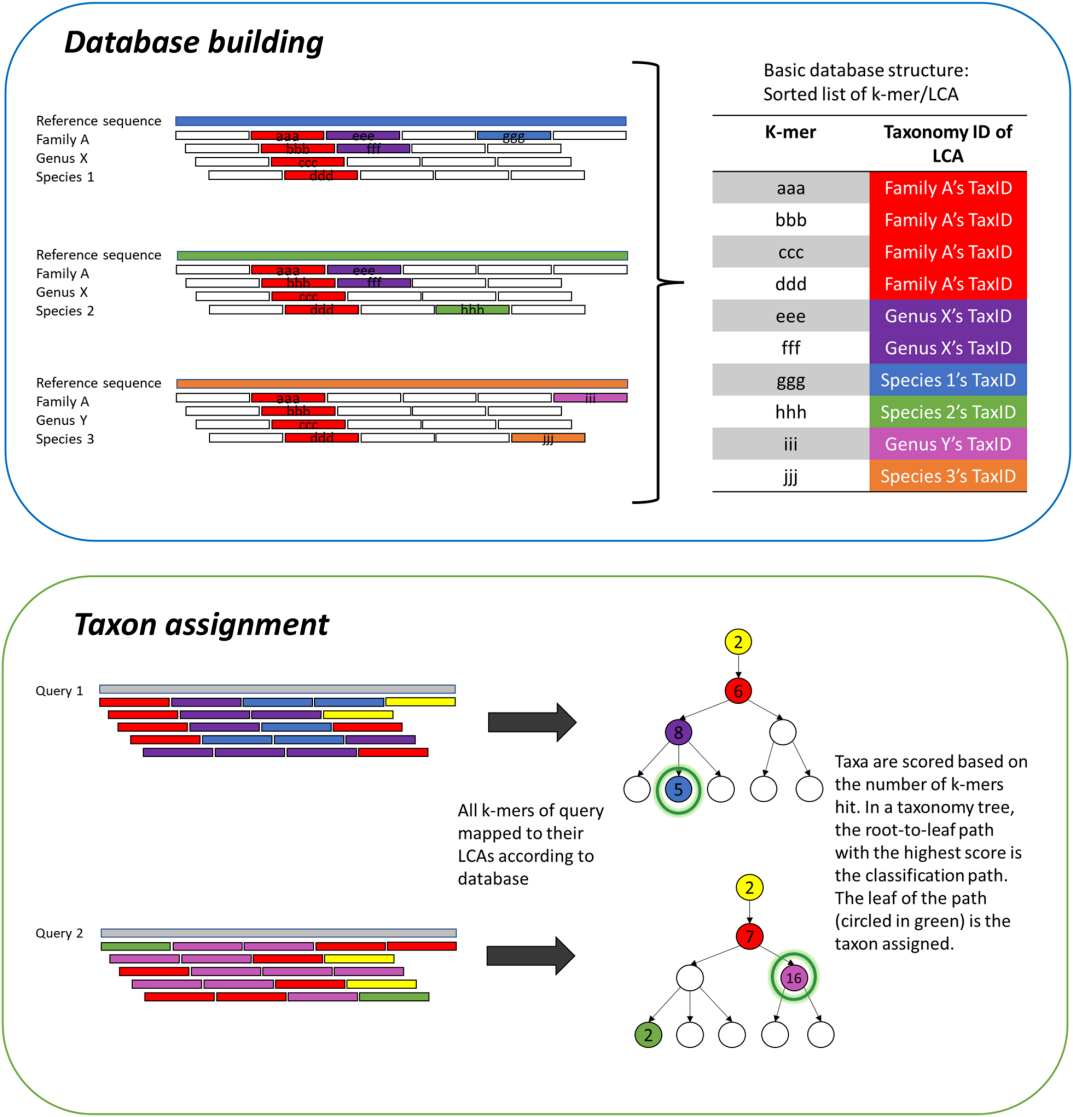
Overview of database building and taxon assignment of Kraken

**Fig. 4:**
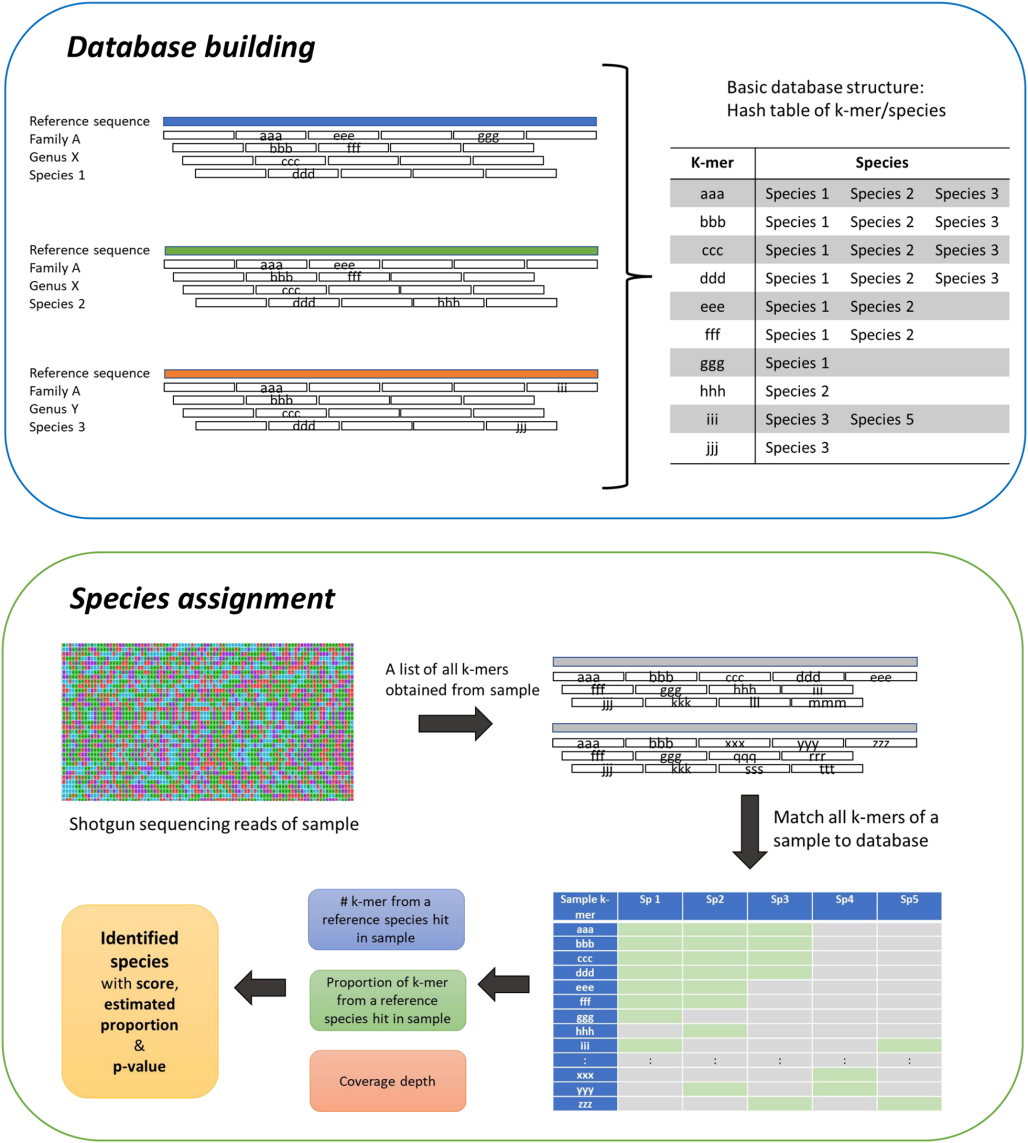
Overview of database building and species assignment of Genome2-ID

The massive raw sequencing reads should be cleaned by removing low-quality reads and/or short reads with length lower than a certain number of bases. Quality-controlled clean reads could be classified by comparing against the reference databases using four different classification approaches: (1) Classical alignment-based method like BLAST and MegaBLAST [51], (2) Burrows-Wheeler transform-based mapping like BWA-MEM [52], Bowtie2 [53] and Centrifuge [54], (3) k-mer-based “pseudoalignment” methods like Kraken [55] and Genome2-ID [21], and (4) a machine learning-based, scikit-learn multinomial naive Bayes classifier (classify-sklearn) supported by q2-feature-classifier, a QIIME 2 plugin for taxonomy classification of amplicon sequences [56]. The former three methods have been adopted in various studies of molecular identification of herbal products by NGS, especially the BLAST method [19, 21, 28, 33]. QIIME 2 and q2-feature-classfier, though highly popular for analysis of microbial sequence data, are not commonly used for herbal identification. There are two possible reasons. The first one is that classify-sklearn of q2-feature-classifier is only suitable for amplicon sequencing. The second reason is the requirement of classifier training for each marker gene (barcode)/reference database combination [56]. The training step is computationally expensive, especially when multiple barcodes are needed for differentiation of plants. The principle of taxon assignment by BLAST or mapping is relatively simple. They are based on sequence similarity between the query sequence and the reference sequences in database. But their output results should be interpreted with care, as multiple top hits with identical quality results could be matched to more than one assigned species. The CITESspeciesDetect pipeline has its own set of interpretation guidelines, which mainly involves placing the reads on the lowest common ancestor (LCA) when multiple hits are obtained per read, i.e., downgrading the OTU matched to more than one congeneric species to genus level, or one matched to more than one con-familial genera to family level [57]. Food Authentication from SEquencing Reads (FASER), a recently published bioinformatic pipeline, has a promiscuity filtering that retains only the BLAST hits with the highest bit score and removes matched taxa S when < 10% of BLAST hits matched to taxa S are unique to S [58]. Unfortunately, in some molecular herbal authentication studies, the parameters and results interpretation of BLAST method were not clearly mentioned. Kraken and Genome2-ID are both k-mer based methods that would involve all reference sequences in available, not just sequences of target barcodes, for database building. Principles of database building and classification of Kraken and Genome2-ID are shown in Figs. 3 and 4, respectively. The taxon assignment algorithm of Kraken has also adopted the concept of LCA, while Genome2-ID is mainly for species assignment. Different features of BLAST, Kraken and Genome2-ID are listed in Table 2.

**Table 2:** Comparison of three taxonomic assignment programs previously used in herbal identification

	BLAST	Kraken	Genome2-ID
Method	Alignment-based	k-mer based	k-mer based
Database	Sequences downloaded from GenBank or BOLD	Indexed and sorted list of k-mer/LCA pairs	A hash table of k-mer annotated with reference species the k-mer was observed with
Classification	1. BLAST-based search 2. Sequence assigned to species or the LCA by MEGAN or CITESspeciesDetect pipeline	1. All k-mers of a sequence are mapped to different LCAs according to database 2. Each hit taxon in the classification tree is scored 3. Sequence assigned to the “leaf” (the lowest taxon rank scored) of the highest weighted “tree branch”/path	1. All k-mers of a sample are mapped to different reference species according to database 2. Presence of the mapped reference species in a sample is determined by computing the number of k-mers of the species matched in the sample, the coverage/proportion of k-mers of the species matched and average coverage depth of the species, with statistical analysis to show confidence for presence of the species
Results output	Multiple species assignments for a given read by BLAST, further analyzed to report LCA of the read/contig	LCA of a given read/contig	Species determined to be present in the sample
Advantage	Customizable database Gold standard for taxonomic assignment [61]	Customizable database Less sensitive to structural rearrangements (e.g. inversions) [45] Detection	Customizable database Less sensitive to structural rearrangements (e.g. inversions) [45] Semiquantitative estimation possible (for genome skimming without PCR) [21]
Disadvantage	Computationally demanding and slow Sensitive to structural rearrangements (e.g. inversions) [45]	High memory requirement (improvable with smaller database or more updated versions like Kraken 2)	Not publicly available
Related programs	BLASTN MegaBLAST	Kraken 2 KrakenUniq Bracken	N/A

In an attempt to compare the classification approaches, Harbert [36] classified a simulated data set using MegaBLAST 2.2.26, Centrifuge 1.0.3-beta and Kraken 0.10.6-unreleased, with reference sequences from all plant (Viridiplantae) taxa in GenBank for building reference databases. It was concluded that Centrifuge had the highest sensitivity, i.e., true positive rate, while Kraken was more conservative with the highest precision, i.e., fewest false positive. MegaBLAST required much higher computation time than the two other methods. The time MegaBLAST required was 100–10,000 times of (two to four orders of magnitude more than) that of Kraken and Centrifuge on the same data sets. Recently, Raine et al. proposed the identification of taxon-specific k-mers and have shown its applicability on tomato plant [59] and *Lupinus* species [60]. Basically, they generated taxa-specific k-mers by removing k-mers existing in all available complete plastid genome sequences from the list of k-mers generated from plastid genome sequences of the target species. However, a MegaBLAST search against all plant (Viridiplantae) taxa except for *Lupinus* revealed that out of the first 200 *Lupinus* spp.-specific k-mers published, 53 of them could be found in other plants with 100% identity (data not shown). This could be due to the deposition of new chloroplast complete genomes in GenBank, or the random occurrence of those “specific” k-mers in nuclear or mitochondrial genomes of other plants. Another concern for the reliability of this approach is the absence of complete chloroplast genome data of most species in GenBank [36]. As of 7 Dec, 2021, there are a total of 238,669 green plant species (Viridiplantae) in NCBI Taxonomy, but only 10,233 of them have their complete chloroplast genome sequences deposited in GenBank. With the increase in complete chloroplast genomes deposited, the previously identified taxon-specific k-mers would be no longer reliable and would require, at the very least, constant updating.

## Qualitative or quantitative, that is the question

Since the development of NGS, it has been tempting to explore whether NGS can provide not just qualitative, but also quantitative or semi-quantitative results. Several studies on the molecular identification of herb or food samples by metabarcoding [62, 63] or genome skimming [21] have also looked into whether the proportion of reads assigned, from either experimental or simulated data with known original proportion, truly reflect the proportion of the species. The results are, in general, inconclusive. In one metabarcoding study, there was a strong correlation between expected and observed quantities of fruits in three fruit mixtures, but not in the other two mixtures [63]. This is not surprising as there would be a variable number of template-primer mismatches across different target species, causing PCR bias. Using an in silico model simulating the post-PCR relative species abundance with 15 COI primer pairs and mitogenomes of 1200 insect species from Refseq of GenBank, Piñol et al. [64] showed that the number of template-primer mismatches and the characteristics of species mixture are important factors determining whether the metabarcoding results would be quantitative. They recommended five primer pairs for insect metabarcoding in general. Their results helped explaining the contradictory conclusions of whether DNA metabarcoding can be quantitative for food and herbal materials. Adaptation of similar in silico modelling followed by in vivo experiments with selected primer pairs and mock reference samples would be a good strategy for establishing an accurate, repeatable and semi-quantitative DNA metabarcoding method for quality control of a specific multi-herb product. For genome skimming, Genome2-ID software is said to be able to achieve semiquantitative estimates of species proportions in products achieved by calculating the number of k-mers matched to a species reference, the coverage, i.e., the proportion of the species matched by the sample data, and the depth, which is the average number of times a k-mer of the species reference was matched by the sample data [21]. A simulated combination of whole genome sequencing reads of *Echinacea purpurea* and *O. sativa* (rice) at a ratio of 99:1 was estimated to be 94:5. It should be noted that the term “quantitative” here only means a significant linear correlation between the relative DNA concentration of the DNA extract before PCR and the proportion of assigned reads after NGS (for DNA metabarcoding), or a significant linear correlation between the relative DNA concentration of the DNA extract and the proportion of assigned reads after NGS (for genome skimming). If we look into “quantification” in a broader sense, i.e., if there is a significant linear correlation between the proportion of assigned reads and the amount (dry weight) of each ingredient, there should be estimation biases introduced in almost each step in Fig. 1. Some of the factors that could potentially influence the species identification results and their quantifiability are listed below.

Raw plant materials

- *Parts of plants* Medicinal herbs sourced from leaves of a plant would naturally contain more chloroplasts, and more chloroplast DNA, than herbs sourced from other parts of a plant.
- *DNA copy number* DNA barcodes may have different copy number in different species and different parts of plants. ITS2, a popular DNA barcode for plants, is a non-coding region in the nuclear ribosomal DNA (rDNA) cluster. It has long been known that the number of rDNA copy in plants varied from 500 to 40,000 per diploid cell [65]. DNA copy number can also be subject to growing conditions. A 5-day dark-induced senescence of *Arabidopsis* leaf could cause a drastic decrease of chloroplast DNA copy number to one-fifth [66].

Processing in preparation of decoction slices and production of multi-ingredient products

- *DNA degradation* In a multi-herb product, some decoction slices might have been heavily processed, and some might not. Different degree of processing will lead to different level of DNA fragmentation.

DNA extraction

- *Variation in DNA extraction efficiency* It has been reported that the DNA yield from the same number of pollen grains from three plants varied a lot (up to 290-fold difference) [67]. Given the diverse parts of plants in Chinese herbal medicine, DNA extraction efficiency from different kind of decoction slices would also vary a lot.

PCR bias (for PCR-based metabarcoding)

- Number of template-primer mismatches
- *Use of indexed primers * It has been shown to affect the relative abundance of detected species in COI metabarcoding [68].

Species/taxon assignment

- Accuracy and abundance (coverage) of reference sequence database
- Discriminatory power of the DNA barcode(s) chosen (for metabarcoding and genome skimming/shotgun metagenomics that relies on homology of certain DNA regions for species assignment)

## New concerns emerge with new scientific knowledge

With the reducing sequencing cost and increasing choices of bioinformatics software or pipelines, research and method development on species identification of multi-herb products by NGS would keep growing at a fast pace. Regardless of experimental approach and sequencing platform, barcode sequences from chloroplast, chloroplast genomes, and ITS sequences remain highly popular targets in analysis for taxon assignment because of their discriminatory power and abundance in public databases. The reliability and accuracy of chloroplast and ITS sequences in reference sequence database are essential for the correct taxon assignment of reads in the bioinformatic pipelines. While it has long been known that rDNA, in which ITS1 and ITS2 are located, is a high-copy gene [65], recent development of NGS revealed that there are intragenomic variations in nuclear ribosomal DNA, including ITS sequences, in plants [69–72]. It was estimated that there was a mean of 35 ITS2 variants per species among 178 plant species. Species from different genera were found to share identical ITS2 variants [69]. As individual reference sequences downloaded from GenBank were likely obtained by Sanger sequencing, it may not be able to fully cover all the variants/alleles of multi-copy regions like ITS1 or ITS2. Song and colleagues even found that one minor ITS2 variant in *Eleutherococcus giraldii* was identical to a major variant of *Panax ginseng* [69]. Similar situations were also reported in *Dendrobium* genus, the source of another popular herb, Herba Dendrobii [72]. The existence of minor variant may not be an issue in traditional DNA barcoding, but it may cause false positive identification in the NGS era.

A recent publication raised the alarm on potential misidentification based on chloroplast barcodes or genomes because of the horizontal plastid genome transfer from chloroplast into mitochondrial and nuclear genomes [73]. Mitochondrial DNA fragments that are derived from plastids are known as mitochondrial plastid DNAs (MTPTs). The existence of homologous sequences in chloroplast and mitochondrial genome was first discovered in early 1980s [74]. But this issue could not have been systematically studied without complete chloroplast and mitochondrial genome assemblies. In 2007, Wang et al. analysed the extent of MTPTs in 11 plants, from which they inferred that the transfer of chloroplast DNA to mitochondrial DNA occurred more than 300MYA. They also estimated that trnV(uac)-trnM(cau)-atpE-atpB-rbcL, rbcL gene included, was the oldest MTPT gene cluster. Another group later embarked on a more comprehensive analysis on 73 plant species and reported that MPTPs were only found in seed plants with a high degree of diversity [75]. Among the 39 seed plants containing MTPTs, *Panax ginseng* had the highest proportion of MTPTs in mitochondrial genome, with the total length of MTPTs occupying 8.0% of the mitochondrial genome of *P. ginseng*. However, the question of how MTPTs would affect authentication of botanical ingredients by DNA barcoding has never been raised until recently. Park and colleagues [73] assembled the plastid and mitochondrial genomes of two closely related and commonly mistaken medicinal plants, *Cynanchum wilfordii* and *C. auriculatum*, to look into the matter. It was found that about 35% of the plastid genomes and almost 50% of plastid protein-coding genes, including the complete genic region of matK, had homologous sequences in the mitochondrial genome of the same species. The homologous genes in the plastid and mitochondrial genomes were found to have different nucleotide substitution rates. They further demonstrated a paradox of “species-specific” DNA marker developed merely based on pairwise alignment of chloroplast genome sequences of closely related target and non-target species. In a “species-specific” DNA marker PCR assay, unexpected bands of the intended size could be obtained from non-target species by increasing the number of PCR cycles, which resulted in amplification of MTPT. As the gene copy number of mitochondria is generally much lower than that of chloroplast in plants [76], the presence of MTPTs should not seriously confound species-specific DNA markers or traditional DNA barcoding with amplicons targeting chloroplast genomes. However, MTPTs might be picked up and sequenced by NGS, leading to mis-identification, especially if k-mer-based identification approach is adopted, or if chloroplast sequences and genomes only are included in reference database for taxon assignment. How MTPTs would affect species identification remain to be investigated, perhaps with simulated data set of plant species with assembled mitogenomes and chloroplast genomes. But the feasibility of developing taxon-specific k-mers [59, 60] solely on chloroplast sequences could already be further challenged by the existence of MTPTs.

New analytic methods call for new standard of references. With the growing popularity of carrying out authentication of multi-herb products by NGS and the evidence of intracellular variations of high-copy genes across the nucleus, chloroplast, and mitochondria, it seems that the idea of establishing reference sequence databases using sequences obtained by genome skimming from reference materials, as proposed by Coissac et al. [35], is arduous but scientifically reasonable. Further investigations on the differences in identification results caused by different analytical methods and/or different reference databases, both in silico with simulated data sets and in vivo with authenticated samples, would also be needed, as a suitable reference database compatible to the taxon assignment method is essential to substantiating the applicability of the method.

## Conclusion

Development of next-generation sequencing has revolutionized the field, allowing rapid accumulation of reference chloroplast, mitochondrial and genomic reference sequences, as well as high-throughput sequencing and species identification of multi-ingredient herbal products or formulations. The experimental workflow of NGS is quite simple, from DNA extraction, library building to high-throughput sequencing. However, there are many factors that could affect the applicability and differentiation power of a NGS experiment, including but not limited to DNA degradation during manufacturing process, differentiation power of DNA barcodes chosen, PCR bias, applicability of taxon assignment program and coverage of reference sequence database. In this review, important considerations for experimental design of NGS for herbal identification have been discussed. Intragenomic heterogeneity of ITS sequence and the presence of mitochondrial plastid DNA were also highlighted to show the necessity of constant updating of reference sequence database and bioinformatics pipeline. We hope that this review provides some guidance on designing and evaluating NGS-based identification for pharmacovigilance or quality assurance of herbal products. NGS has allowed simultaneous identification of not only the expected ingredients, but also contaminants and adulterants. However, DNA-based method cannot identify the parts of plants/animals used in the products, nor can it identify the chemical components qualitatively or quantitatively. Quality control of herbal products involves various aspects, which cannot be comprehensively evaluated by any standalone assays. To ensure the identity and quality of herbal products for the benefit of the industry and consumers, other independent technologies, such as chemometric-guided profiling [77], biological evaluation and metabolomics [78], should also be employed in an integrative manner.

## Data Availability

Not applicable.

## Abbreviations

16S: 16S ribosomal RNA gene
BLAST: Basic Local Alignment Search Tool
BOLD: Barcode of Life Data System
BWA: Burrows-Wheeler-Alignment Tool
CCS: Circular consensus sequencing
COI: Mitochondrial cytochrome oxidase subunit 1 gene
DNA: Deoxyribose nucleic acid
dPCR: Digital polymerase chain reactions
EBC: Extraction blank control
EPC: Extraction positive control
FASER: Food Authentication from SEquencing Reads
ITS2: Internal transcribed spacer 2
LCA: Lowest common ancestor
MPTPs: Mitochondrial plastid DNAs
NGS: Next-generation sequencing
OTU: Operational taxonomic unit
PCR: Polymerase chain reactions
psbA-trnH: Intergenic spacer between Photosystem II protein D1 gene and tRNA-His gene
QIIME: Quantitative Insights Into Microbial Ecology
qPCR: Real-time quantitative polymerase chain reactions
rbcL: Ribulose bisphosphate carboxylase large chain
SMRT: Single-Molecule Real-Time
trnL: tRNA-Leu gene
